# Characteristics and Help-Seeking Behaviors of Internet Gamblers Based on Most Problematic Mode of Gambling

**DOI:** 10.2196/jmir.3781

**Published:** 2015-01-07

**Authors:** Nerilee Hing, Alex Myles Thomas Russell, Sally Melissa Gainsbury, Alex Blaszczynski

**Affiliations:** ^1^Centre for Gambling Education and ResearchSouthern Cross UniversityLismoreAustralia; ^2^Gambling Treatment Clinic & ResearchThe University of SydneySydneyAustralia

**Keywords:** gambling, Internet, pathological gambling, treatment

## Abstract

**Background:**

Previous studies of problem Internet gamblers have failed to distinguish whether their problem gambling relates to Internet or land-based gambling modes. Therefore, characteristics and help-seeking behaviors of people whose gambling problems relate specifically to Internet gambling are unknown, but could inform the optimal alignment of treatment and support services with the needs and preferences of problem gamblers.

**Objective:**

This study aimed to compare (1) characteristics of problem Internet gamblers and problem land-based gamblers and (2) uptake of different types and modes of help between problem Internet gamblers and problem land-based gamblers. Hypothesis 1 was that problem Internet gamblers are less likely to seek help. Hypothesis 2 was that problem Internet gamblers are more likely to use online modes of help.

**Methods:**

A sample of 620 respondents meeting criteria for problem gambling was drawn from an online survey of 4594 Australian gamblers. Respondents were recruited through advertisements on gambling and gambling help websites, Facebook, and Google. Measures consisted of gambling participation; proportion of gambling on the Internet; most problematic mode of gambling; help seeking from 11 different sources of formal help, informal help, and self-help for gambling problems; psychological distress (Kessler 6); problem gambling severity (Problem Gambling Severity Index, PGSI); and demographics.

**Results:**

Problem Internet gamblers were significantly more likely than problem land-based gamblers to be male (χ^2^_1_=28.3, *P*<.001, φ=0.21), younger (*t*_616.33_=4.62, *P*<.001, *d*=0.37), have lower psychological distress (χ^2^_1_=5.4, *P*=.02, φ=0.09), and experience problems with sports and race wagering (χ^2^_4_=228.5, *P*<.001, φ=0.61). Uptake of help was significantly lower among problem Internet compared to problem land-based gamblers (χ^2^_1_=6.9, *P*<.001, φ=0.11), including from face-to-face services, gambling helplines, online groups, self-exclusion from land-based venues, family or friends, and self-help strategies. Both problem Internet and problem land-based gamblers had similarly low use of online help. However, problem land-based gamblers (37.6%, 126/335) were significantly more likely to have sought land-based formal help compared to problem Internet gamblers (23.5%, 67/285; χ^2^_1_=14.3, *P*<.001, φ=0.15).

**Conclusions:**

The findings suggest that more targeted and innovative efforts may be needed to increase use of gambling help by problem Internet gamblers. Alternatively, their lower PGSI and K6 scores suggest Internet problem gamblers may have less need for gambling-related help. This is the first known study to classify problem Internet gamblers as those whose problem gambling specifically relates to Internet gambling. Further research is needed to better understand why help-seeking rates are lower among Internet problem gamblers.

## Introduction

### Background

Problem gambling is characterized by difficulties in limiting time and/or money spent on gambling which leads to adverse consequences for the gambler, others, or the community [1]. Problem gamblers are typically distinguished by a pattern of excessive gambling, impaired control over gambling, and persistence with heavy gambling despite its significant negative consequences [2]. Prevalence studies in 202 jurisdictions indicate past-year problem gambling rates of 0.5%-7.6% of the adult population [3]. Severe negative financial, relationship, health, vocational, and legal consequences of problem gambling, along with low help-seeking rates, suggest that further research into the disorder and associated help seeking is warranted.

Although use of Internet gambling is increasing internationally and several studies have examined Internet gamblers, little is known about their preferences and likelihood of seeking help for gambling problems. This knowledge is lacking because most studies have classified problem Internet gamblers as Internet gamblers who meet criteria for problem gambling, regardless of whether their problem gambling is related to Internet or land-based gambling modes [4-11]. Therefore, this classification would identify a person as a problem Internet gambler who has significant problems with gambling on land-based table games, wagering, or electronic gaming machines (EGMs, also known as slot machines, poker machines, video slots, and fruit machines), but who occasionally purchases a lottery ticket online. Accordingly, this approach has been able to draw only limited conclusions about the role of Internet gambling in problem gambling and characteristics and help-seeking behaviors of people whose gambling problems relate specifically to online gambling modes.

The present study aimed to (1) compare the characteristics of problem Internet gamblers to problem land-based gamblers, differentiated according to their most problematic mode of gambling and (2) compare the uptake of different types and modes of help between problem Internet gamblers and problem land-based gamblers.

To our knowledge, this study is the first with a nonclinical sample to classify problem Internet gamblers as individuals whose problem gambling is specifically associated with Internet gambling. Understanding the comparative similarities and differences in characteristics and help-seeking behaviors of those nominating Internet (vs land-based) gambling modes can guide the optimal alignment of treatment and support services with the needs and preferences of problem Internet gamblers. This should improve rates of gambling help seeking from their currently low base rate. This is relevant given only 5%-10% of problem gamblers are in professional treatment at any 1 time and only small proportions access other supports such as self-exclusion (in which people voluntarily bar themselves from gambling venues or websites for a specified time period), peer support groups, informal assistance from family and friends, and self-help [12-20].

### Characteristics and Help-Seeking Behavior of Problem Internet and Land-Based Gamblers

Research has not yet established whether the help-seeking behavior of problem Internet gamblers differs from that of problem land-based gamblers because the most problematic mode of gambling has not been considered previously. However, several reasons are discussed subsequently that support the 2 hypotheses tested in this study:

1. Hypothesis 1: Problem Internet gamblers are less likely to seek help for problem gambling compared to problem land-based gamblers.
2. Hypothesis 2: Problem Internet gamblers are more likely to seek online help for problem gambling compared to problem land-based gamblers.

Our first hypothesis was proposed based partly on the different sociodemographic and psychological profile of problem Internet gamblers compared to problem land-based gamblers. A large representative Australian telephone survey conducted in late 2011 (with different participants than this study) found that Internet moderate-risk/problem gamblers were more likely to be younger, male, married, and to have lower levels of psychological distress compared to moderate-risk/problem land-based gamblers [4]. Another large study conducted in 2006-2007 with Canadian and international samples [21] found that problem Internet gamblers were more likely to be single, of Asian ancestry, with lower household income, and have mental health problems and a history of other addictions. In a small sample of Spanish treatment-seeking problem gamblers recruited between 2005 and 2009, those who gambled only online (n=53) tended to have higher education and socioeconomic status than problem land-based gamblers [22]. Previous studies have found that help-seeking for problem gambling is less common among problem gamblers who are male, younger, unmarried, employed, and in ethnic minority groups [14,23-28], which largely aligns with the general profile of problem Internet gamblers, although this varies by jurisdiction. Thus, problem Internet gamblers in the current study were expected to be less likely to seek help than their land-based counterparts.

Differences in types of problematic gambling between Internet gamblers and land-based gamblers also lend support for our first hypothesis that problem Internet gamblers are less likely to seek help for problem gambling. An Australian telephone survey found that Internet problem gamblers were more likely to experience problems with sports and race wagering, and land-based problem gamblers were more likely to experience problems with EGMs [4]. Another study found Canadian Internet gamblers were significantly more likely to report poker as their most problematic gambling form compared to EGMs for non-Internet gamblers [11]. Although there is widespread agreement that most problem land-based gamblers attribute their problems to EGMs [13,17], problematic Internet gambling forms appear to be more diverse and far less commonly related to online EGM play. EGMs are played mostly in land-based venues where gambling help services are often widely advertised; in contrast, gambling on sports, races, and poker is more likely to occur through online websites where the advertising of help services is generally less apparent. Thus, problem Internet gamblers may be less likely to seek help because they are less exposed to gambling help service advertising compared to problem land-based gamblers.

Other features of Internet gambling may also impede help seeking for problem gambling. Use of electronic money, accounts, and credit may delay problem recognition and acknowledgment in contrast to land-based cash gambling where losses are immediately apparent [5,29]. Lack of scrutiny in the online environment may also facilitate problem denial, whereas staff and other patrons in land-based venues may heighten a gambler’s attention to the extent of their gambling, either overtly or implicitly [29]. These distinctive features of Internet gambling can be expected to result in lower help-seeking rates.

Further, the negative consequences of gambling appear to differ between problem Internet and problem land-based gamblers. One study found that moderate-risk/problem land-based gamblers are more likely to experience more serious gambling consequences than their Internet gambler counterparts [30]. These consequences included major relationship breakdown, loss of contact with children, change or loss of employment, bankruptcy, and loss of savings. Given that help seeking is typically crisis-driven [13,31], less extensive, and severe, negative gambling consequences may lead to lower uptake of help by problem Internet gamblers compared to problem land-based gamblers.

In support of our second hypothesis that problem Internet gamblers are more likely to seek online help for problem gambling compared to problem land-based gamblers, preferred mode of help (face-to-face, telephone, online) may align with preferred mode of gambling. Studies suggest that Internet gamblers more frequently access Internet-based help and this may be a preference. Wood and Griffiths’ [32] 2007 evaluation of a UK-based online help service for problem gamblers found that online gambling was the most popular gambling “location” among clients and more popular than among clients accessing a comparable UK gambling telephone helpline. The authors argued that online help is most likely the preferred mode for online gamblers because of their likely greater overall competence in, familiarity and comfort with, and access to the Internet. A 2009 study of 2 UK online forums for people affected by gambling problems also found they were most popular with online gamblers [33]. Of 2021 clients accessing real-time chat and email support through an Australian online gambling help service between 2009 and 2011, 16.6% preferred an online gambling mode [34], more than double the proportion of Internet gamblers in the Australian adult population [4]. Rodda and Lubman [34] speculated that the same reasons for preferring online gambling, such as convenience, ease, and comfort of their own home, may also make online help more attractive to this group. However, 1 large study found that both Internet and non-Internet problem/moderate-risk gamblers in its Canadian (n=8948) and international (n=12,521) samples expressed a preference for using a face-to-face service over an online or telephone service [11].

## Methods

### Overview

Approval for this study was obtained from 2 university human research ethics committees (Southern Cross University, The University of Sydney). An online survey was used to reach a large national sample, and for its anonymity and privacy which enhance response rates, response accuracy, and survey completeness, especially when focused on sensitive topics such as gambling [21,35]. Although the survey was only available to those with Internet access, 82.3% of Australians are Internet users [36].

### Recruitment and Sampling

Advertisements were placed on 46 websites likely to be visited by Australians interested in gambling and obtaining help for gambling problems. These websites included those of 18 regulated gambling operators, 12 gambling-related sites (containing information and research), 7 government departments which regulate gambling, 6 gambling help organizations, and 3 sporting associations. Paid advertisements were also displayed on Facebook and Google. A sample of 4594 respondents was obtained between May and December 2012. Inclusion criteria were aged 18 years or older, gambling at least once in the previous 12 months, and English literacy. Most respondents (53.87%, 2475/4594) were alerted to the survey via advertisements on online wagering/lottery sites, Facebook (17.63%, 810/4594), and Google (6.27%, 288/4594).

### Measures

#### Gambling Behavior

Gambling behavior was measured, including participation during the previous 12 months in 10 gambling forms: instant scratch tickets; lottery, lotto, or pools tickets; sports betting; betting on horse or dog races; bingo; keno; poker; casino table games not including poker; games of skill not including poker; and EGMs. For each type of gambling, respondents were also asked the percentage of their “purchases or play” during the previous 12 months that “was done over the Internet.” Global measures to assess the proportion of all gambling money and time spent online were also included. Two questions were asked about use of alcohol and use of recreational drugs while gambling, with response options from “never” to “almost always.”

#### Problem Gambling Severity

The Problem Gambling Severity Index (PGSI) [37] is widely used in Australia and elsewhere and is a recommended measure of problem gambling severity [38]. This 9-item scale is scored “never” (=0) to “almost always” (=3), with scores summed for a total between 0-27. Cut-off scores adhered to those used in the PGSI’s original validation in which 0=nonproblem gambler, 1-2=low-risk gambler, 3-7=moderate-risk gambler, and 8-27=problem gambler. Cronbach alpha for this scale in this sample was .93. Only respondents scoring between 8 and 27 were included in the current study because those at lower risk, such as moderate-risk gamblers, tend not to seek help for a gambling problem [38].

#### Most Problematic Form of Gambling

Problem gamblers (based on PGSI scores) were asked which form of gambling had contributed most to any problems that they had experienced from gambling. Respondents could choose 1 of 10 forms listed previously, “other,” or “I have not experienced problems from my gambling.”

#### Most Problematic Mode of Gambling

Problem gamblers (based on PGSI scores) were asked “What type of gambling medium has contributed MOST to any problems you may have experienced from your gambling?” The following response options were provided: Internet via computers, Internet via mobile phone, Internet via portable/wireless device, Internet TV, land-based gambling, and betting via telephone. The first 4 options were recoded into “Internet gambling” to classify most problematic gambling mode.

#### Help Seeking for Gambling Problems

Problem gamblers were asked if they had ever sought help from 11 different sources encompassing the most common forms of formal help, informal help, and self-help [18]. Formal types of help were grouped for some analyses according to mode of help: (1) land-based forms (face-to-face from a specialist gambling counselor, face-to-face from a nongambling specialist professional, face-to-face support group, residential treatment program, self-exclusion from a land-based gambling venue), (2) online modes (online or email gambling counseling, online support group or discussion board, self-exclusion from a gambling website), and (3) telephone modes (gambling telephone helpline). In Australia, specialized gambling help services are provided free through government-funded services. These include appointment-based face-to-face counseling and immediate 24/7 information, support, counseling, and referral through telephone and online services. However, people may also access help through private practitioners (may incur a cost).

#### Psychological Distress

Psychological distress was assessed by the 6-item Kessler Psychological Distress Scale (K6) [39], which asked frequency of symptoms of psychological distress over the most recent 4 weeks with fixed responses ranging from “none of the time” (=0) to “all of the time” (=4). Total scores of 12-19 indicate mild to moderate mental health disorders and scores greater than 20 indicate clinically high levels of psychological distress [40,41]. The scale exhibited good reliability in this sample (Cronbach alpha=.93).

#### Demographics

Sex, age, place of residence, household type, highest educational qualification, work status, income, debt, and cultural background were self-reported.

### Analyses

Problem Internet gamblers were defined as those meeting criteria for problem gambling as measured by the PGSI [37] and who nominated the Internet as their most problematic gambling mode. Problem land-based gamblers were defined as those meeting PGSI criteria for problem gambling and who nominated a land-based gambling mode as their most problematic. Most statistical analyses compared these 2 groups of respondents. These comparisons took the form of nonparametric tests (Mann-Whitney *U*), chi-square tests of independence (with post hoc pairwise comparisons using *z* tests for multiple degrees of freedom tests), or independent samples *t* tests. A binary logistic regression was also conducted and this is detailed in the results section. Finally, repeated measures nonparametric analyses were conducted for expenditure using the Wilcoxon signed rank test. Nonparametric tests were used for ordinal frequency data or for expenditure data where extreme values resulted in excessive variance. Alpha was set at .05 for all analyses unless stated otherwise.

## Results

### Sample Characteristics

Of the 4594 respondents to the survey, 70.51% (3239/4594) had gambled using the Internet at least once during the previous 12 months (designated as Internet gamblers), whereas 29.49% (1355/4594) had gambled only on land-based modes (designated as land-based gamblers). Among the 4594 respondents, 643 scored as problem gamblers on the PGSI, representing 14.20% (460/3239) of Internet gamblers and 13.51% (183/1355) of land-based gamblers in the sample. Problem land-based gamblers exhibited significantly higher PGSI scores (mean 14.4, SD 5.3, median 13.0) compared to problem Internet gamblers (mean 12.5, SD 4.6, median 11.0; *U*=37,845.5; *z*=4.47, *P*<.001).

Of the 643 problem gamblers surveyed, 9 respondents listed telephone betting as their most problematic medium and were excluded from further analysis, as were 14 people who did not answer the question. Of the remaining 620 problem gamblers, 285 (46.0%) nominated Internet modes as their most problematic either via computers, mobile phones, portable/wireless devices or interactive television (designated as problem Internet gamblers). The remaining 335 respondents (54.0%) nominated land-based modes as their most problematic (designated as problem land-based gamblers). Thus, problem Internet gamblers were past-year problem gamblers who nominated an Internet gambling medium as contributing most to their gambling problems. Problem land-based gamblers were past-year problem gamblers who nominated a land-based gambling medium as contributing most to their gambling problems.

The sample of problem gamblers (n=620) was predominantly male (79.8%, 495/620), with a mean age of 37.6 years (SD 13.1). The most common marital status was never married (44.4%, 275/620) followed by married (25.6%, 159/620) or de facto/living with partner (17.9%, 111/620). At least 70.0% (434/620) of the sample reported living with others and 62.6% (388/620) reported living in a major metropolitan city. Most (53.5%, 332/620) worked full-time and 52.7% (327/620) reported completing some form of tertiary study. Most (79.0%, 490/620) were born in Australia and 81.0% (502/620) spoke English at home as their primary language. Median household income was AU$60,000-AU$69,999 per annum, with median household debt reported to be AU$20,000.

### Characteristics of Problem Internet vs Land-Based Gamblers

#### Overview

Problem Internet gamblers were compared to problem land-based gamblers for demographic characteristics, mental health, gambling behavior, and most problematic gambling form.

#### Demographic Characteristics

Problem Internet gamblers were significantly more likely to be male (89.1%, 254/285) compared to problem land-based gamblers (71.9%, 241/335; χ^2^_1_=28.3 *P*<.001; φ=0.21) and significantly younger (mean 35.0, SD 11.3) compared to problem land-based gamblers (mean 39.7, SD 14.0; *t*_616.33_=4.62, *P*<.001; *d*=0.37).

No significant differences were observed between problem Internet and problem land-based gamblers for any of the following demographic variables: marital status, household characteristics (ie, number of children or absence/presence of partners), location of residence (approximately 62% of both groups lived in major metropolitan cities), level of education, work status, household income or debt, country where the respondent was born, or language spoken at home.

#### Mental Health

Problem land-based gamblers were significantly more likely to be classified as having high psychological distress according to the K6 than were problem Internet gamblers (37.6%, 126/335 vs 28.8%, 82/285, respectively; χ^2^_1_=5.4, *P*=.02; φ=0.09).

#### Gambling Participation, Frequency, Expenditure, and Substance Use While Gambling

A significantly higher proportion of problem Internet gamblers participated in sports betting and horse and dog race betting, whereas a significantly higher proportion of problem land-based gamblers took part in EGMs, with no significant differences for lottery-type or other forms of gambling (Table 1). Furthermore, problem Internet gamblers who took part in sports or horse race betting did so more often (*U*=16,002, *z*=6.52, *P*<0.001 and *U*=16,461.5, *z*=5.50, *P*<.001, respectively) and spent more money (*U*=18,319.5, *z*=3.45, *P*<.001 and *U*=18,351, *z*=2.82, *P*<.001, respectively) on those forms than did problem land-based gamblers. In contrast, problem land-based gamblers gambled on EGMs significantly more frequently (*U*=17,685.5, *z*=7.20, *P*<.001) and spent significantly more money per month on them (*U*=14,248.5, *z*=8.46, *P*<.001) than did problem Internet gamblers. Repeated measures Wilcoxon signed rank tests were used to compare these 3 forms of betting for expenditure (reported here as *z* tests). Problem Internet gamblers spent more money on horse or dog race betting than sports betting (*z*=2.32, *P*=.02) or on EGMs (*z*=4.58, *P*<.001), but there was no significant difference between sports betting and EGMs (*z*=1.15, *P*=.25). Problem land-based gamblers reported spending significantly more money on EGMs than on horse or dog racing (*z*=5.35, *P*<.001) or sports betting (*z*=7.21, *P*<.001), and more on horse or dog race betting than on sports betting (*z*=3.55, *P*<.001). No significant differences were found between the 2 groups for alcohol or drug consumption while gambling.

**Table 1:** Number and percentage of respondents who participated in each gambling form in the past 12 months by most problematic gambling mode (N=620).

Form	Problem Internet gamblers (n=285)		Problem land-based gamblers (n=335)		χ^2^_1_	*P*	φ
	n (%)^a^	% Online^b^	n (%)^a^	% Online^b^			
Sports betting	248 (87.0)	72.6	198 (59.1)	35.8	59.4	<.001	0.31
Horse and dog race betting	223 (78.2)	70.1	210 (62.7)	28.8	17.7	<.001	0.17
EGMs	186 (65.3)	10.0	307 (91.6)	4.4	65.8	<.001	0.33
Lottery-type gambling^c^	258 (90.5)	NA	308 (91.9)	NA	0.4	.53	0.03
Other^d^	166 (58.2)	NA	186 (55.5)	NA	0.5	.50	0.03

^a^The percentages refer to the percentage of respondents in each group who reported engaging in that form of gambling during the past 12 months. Multiple responses allowed. The statistics are based on these values.

^b^ The percentages refer to the mean percentage of that activity reported by respondents in each group as being conducted via an Internet mode (vs a land-based mode). These cannot be calculated for the lottery-type or other forms because they are combinations of numerous forms (NA: not applicable).

^c^ Lottery-type gambling includes instant scratch tickets, lotteries/lotto/pools tickets, bingo, and keno.

^d^ Other forms include poker, casino table games, games of skill, and any other forms. Multiple answers possible.

#### Most Problematic Gambling Form

When asked about the form of gambling that had most contributed to their gambling problems, problem Internet gamblers were significantly more likely to nominate sports betting and horse or dog race betting compared to problem land-based gamblers, who were significantly more likely to nominate EGMs (χ^2^_4_=228.5 *P*<.001; φ=0.61) (Table 2).

**Table 2:** Number and percentage of respondents who attributed their gambling problems to each form by most problematic gambling mode (N=620).

Form	Problem Internet gamblers (n=285), n (%)	Problem land-based gamblers (n=335), n (%)
Sports betting	82 (28.8)	8 (2.4)
Horse and dog race betting	107 (37.5)	34 (10.1)
EGMs	51 (17.9)	248 (74.0)
Lottery-type gambling^a^	17 (6.0)	10 (3.0)
Other^b^	28 (9.8)	35 (10.4)

^a^Lottery-type gambling includes instant scratch tickets, lotteries/lotto/pools tickets, bingo, and keno.

^b^Other forms include poker, casino table games, games of skill, and any other forms.

### Uptake of Different Types and Modes of Help by Problem Internet vs Problem Land-Based Gamblers

#### Overview

Among the 620 problem gamblers, 342 (55.2%) reported having sought at least 1 type of help for their gambling. Among the problem Internet gamblers, 141 of 285 (49.5%) had sought at least 1 type of help compared to 201 of 335 (60.0%) problem land-based gamblers. This difference was statistically significant (χ^2^_1_=6.9, *P*<.001; φ=0.11) and supported our first hypothesis that problem Internet gamblers are less likely to seek help for problem gambling.

#### Uptake of Different Types of Help

Table 3 compares uptake of the 11 different types of help between problem Internet and problem land-based gamblers. Problem land-based gamblers were significantly more likely than problem Internet gamblers to seek help from the following sources: face-to-face from a specialist gambling counselor, face-to-face from a nongambling specialist professional, gambling telephone helpline, an online support group or discussion board, through self-exclusion from a land-based gambling venue, from family or friends, and through self-help strategies. Problem land-based gamblers were also significantly more likely to have sought any type of help compared to problem Internet gamblers, providing further support for our first hypothesis that problem Internet gamblers are less likely to seek help for problem gambling.

**Table 3:** Number and percentage of respondents who sought each form of help by most problematic gambling mode (N=620).^a^

Form		Problem Internet gamblers (n=285), n (%)	Problem land-based gamblers (n=335), n (%)	χ^2^_1_	*P*	φ
**Formal help**						
	Face-to-face from a specialist gambling counselor	37 (13.0)	77 (23.0)	10.3	.001	0.13
	Face-to-face from a nongambling specialist professional	23 (8.1)	44 (13.4)	4.1	.04	0.08
	Gambling telephone helpline	35 (12.3)	66 (19.7)	6.2	.01	0.10
	Online or email gambling counseling	27 (9.5)	31 (9.3)	<0.1	.93	<0.01
	Residential treatment program	7 (2.5)	6 (1.8)	0.3	.57	0.02
	Face-to-face support group	16 (5.6)	31 (9.3)	2.9	.09	0.07
	Online support group or discussion board	3 (1.1)	12 (3.6)	4.2	.04	0.08
	Self-exclusion from land-based gambling venue	8 (2.8)	54 (16.1)	30.3	<.001	0.22
	Self-exclusion from gambling website	21 (7.4)	19 (5.7)	0.7	.39	0.03
**Informal help**						
	From family or friends	32 (11.2)	73 (21.8)	12.2	<.001	0.14
**Self-help**						
	Through self-help strategies	33 (11.6)	89 (26.6)	21.9	<.001	0.19
	Never sought gambling help	144 (50.5)	134 (40.0)	6.9	<.001	0.11

^a^Multiple responses were accepted.

#### Uptake of Different Modes of Help

Formal sources of help were categorized into land-based modes, online modes, and telephone modes to test our second hypothesis. A total 193 of 620 problem gamblers (31.1%) had sought formal land-based help, 103 of 620 (16.6%) had sought formal online help, and 101 of 620 (16.3%) had sought formal telephone help. There was some overlap between land-based, online, and telephone help seeking (Table 4). All comparisons between those who did and did not seek each form of help were treated independently. To account for any overlap, alpha was set at .01 for the following results.

**Table 4:** Combinations of modes of formal help seeking.

Form(s)	n (%)
No formal help	335 (54.0)
Land-based help only	110 (17.7)
Online help only	49 (7.9)
Telephone help only	35 (5.6)
Land-based and online help	25 (4.0)
Land-based and telephone help	37 (6.0)
Online and telephone help	8 (1.3)
All 3 forms	21 (3.4)

#### Mode of Formal Help by Participant Characteristics

Those who sought land-based help were significantly older (mean 40.1, SD 13.1 years) compared to those who had not sought land-based help (mean 36.4, SD 12.9 years; *t*_618_=3.25, *P*=.001; *d*=0.26), but there was no difference in age between those who had and had not sought online help or telephone help.

No significant differences were observed between those who had and had not sought any of the 3 modes of help by the following demographic variables: gender, marital status, household characteristics, location of residence, level of education, work status, household income or debt, country where the respondent was born, or language spoken at home.

#### Mode of Formal Help Sought by Most Problematic Gambling Mode

Problem land-based gamblers (37.6%, 126/335) were significantly more likely to have sought land-based formal help compared to problem Internet gamblers (23.5%, 67/285; χ^2^_1_=14.3, *P*<.001; φ=0.15). However, no significant differences between the groups were observed for use of online help (16.8%, 48/285 of problem Internet gamblers vs 16.4%, 55/335 of problem land-based gamblers) or for use of telephone help (12.3%, 35/285 for problem Internet and 19.7%, 66/335 for problem land-based gamblers). Therefore, our second hypothesis that problem Internet gamblers are more likely to seek online help for problem gambling compared to problem land-based gamblers was not supported.

### Multivariate Analysis of Types of Help by Most Problematic Gambling Mode

The preceding analyses are univariate analyses. Thus, a multivariate analysis was employed to account for overlap among the results. The dependent variable in this analysis was whether the respondent was a problem Internet gambler (coded as 1) or problem land-based gambler (coded as 2). The predictors were the 11 help-seeking variables (coded as 0=“have not sought this form of help” and 1=“have sought this form of help at least once,” excluding the “never sought gambling help” variable), gender (reference group=female), and age in years (treated as a continuous predictor). The analysis was run as a binary logistic regression with all predictors entered in 1 step. Before conducting the logistic regression, a linear regression was run to check for tolerance issues. The lowest tolerance value obtained was 0.72, indicating little overlap between predictors. Alpha was set to .05 for all predictors.

The overall logistic regression model was significant (χ^2^_13_=99.5, *P*<.001) and successfully predicted 63.9% (182/285) of problem Internet and 64.8% (217/335) of problem land-based gamblers. Results for the predictors are shown in Table 5. Problem land-based gamblers were significantly more likely to have sought help from family or friends, to have attempted self-help strategies, or to have self-excluded from a land-based gambling venue. Problem Internet gamblers were significantly more likely to be male, to be younger, and to have self-excluded from gambling websites. No other variables were statistically significant. Thus, although the tolerance statistics indicate relatively little crossover between the variables, there was enough crossover for these other variables to not be statistically significant in a multivariate procedure.

**Table 5:** Predictors in the logistic regression predicting Internet or land-based problem gambling status by key demographics and forms of help seeking.

Predictor		B (SE)	Wald	*P*	OR (95% CI)
Age (in years)		0.021 (0.007)	8.705	.003	1.021 (1.007, 1.036)
Gender (ref: female)		–0.955 (0.247)	14.981	<.001	0.385 (0.237, 0.624)
**Help-seeking forms (ref: no)**					
	Face-to-face from a specialist gambling counselor	0.379 (0.257)	2.173	.14	1.461 (0.883, 2.418)
	Face-to-face from a nongambling specialist professional	–0.089 (0.332)	0.072	.79	0.915 (0.478, 1.753)
	Gambling telephone helpline	0.312 (0.268)	1.354	.25	1.366 (0.808, 2.308)
	Online or email gambling counseling	–0.514 (0.324)	2.521	.11	0.598 (0.317, 1.128)
	Residential treatment program	–1.310 (0.742)	3.115	.08	0.270 (0.063, 1.156)
	Face-to-face support group	–0.313 (0.421)	0.553	.46	0.731 (0.320, 1.668)
	Online support group or discussion board	0.846 (0.750)	1.273	.26	2.331 (0.536, 10.142)
	From family or friends	0.725 (0.271)	7.132	.008	2.064 (1.213, 3.513)
	Self-exclusion from land-based gambling venue	1.773 (0.443)	16.060	<.001	5.891 (2.475, 14.023)
	Self-exclusion from gambling website	–1.036 (0.410)	6.393	.01	0.355 (0.159, 0.792)
	Through self-help strategies	0.583 (0.269)	4.719	.03	1.792 (1.059, 3.033)

## Discussion

### Principal Results and Comparisons With Prior Work

To our knowledge, this study is the first to identify the characteristics of problem Internet gamblers classified according to most problematic mode of gambling. Consistent with earlier reports, the current study found that problem Internet gamblers were significantly more likely to be male and younger compared to their land-based counterparts [11,42,43]. However, age and gender were found to significantly distinguish problem Internet and problem land-based gamblers in contrast to previous studies in which marital status, ethnicity, socioeconomic status, and education were also implicated [4,21,22]. Our results identified higher rates of psychological distress among problem land-based gamblers compared to problem Internet gamblers, which is the reverse of previous findings [21]. These inconsistencies are likely due to the different method used to classify problem Internet gamblers in this study. In contrast to this study, previous studies have included Internet gamblers experiencing problems with a land-based mode of gambling as problem Internet gamblers [4,5,21].

Our results lend support to previous findings that Internet gamblers are more involved and diverse gamblers compared to non-Internet gamblers [4,11,42,44], at least in their use of different gambling modes. Problem Internet gamblers spent one-fifth of their gambling expenditure and time on land-based gambling modes, whereas problem land-based gamblers spent minimal expenditure and time on Internet gambling modes. As expected from prior research [13,34], EGMs were the most problematic form for land-based gamblers, whereas sports and race wagering were most problematic for problem Internet gamblers.

This study has revealed new information about help seeking among problem Internet gamblers. In support of our first hypothesis, it found that problem Internet gamblers were significantly less likely to access help than their land-based equivalents. This was reflected in their significantly lower uptake of professional face-to-face help and through a gambling helpline, self-exclusion from a land-based venue, from family or friends, and through self-help strategies. Problem Internet gamblers also had lower usage of online support groups or discussion boards compared to problem land-based gamblers. The results provide some indication that problem Internet gamblers were more likely to have self-excluded from a gambling website, but this was the only type of help used more than problem land-based gamblers. This greater use is unsurprising given that online gambling sites created the greatest problems for this cohort. It is possible that website self-exclusion is an adequate intervention for some Internet gamblers to maintain control over their gambling. Nevertheless, less than 1 in 10 problem Internet gamblers had used this intervention.

The most popular types of help among the problem Internet gamblers were face-to-face gambling counseling, followed by a gambling helpline and use of self-help and support from family and friends. Uptake of diverse forms of help may indicate that provision of a wide range of help options best caters for the varying preferences of problem Internet gamblers. However, slightly more than half had never sought any type of help for their gambling. These findings may reflect the greater promotion of help services advertised in land-based venues compared to online gambling websites. As suggested in prior studies, further publicity of formal help services, self-help tools and resources, and encouragement to use family and social support may be needed by online gambling sites to improve help-seeking rates among Internet gamblers [30,34,45-48].

Additional explanations are possible for the lower uptake of help among problem Internet gamblers. Lower uptake may reflect greater reticence to use gambling help among males and younger people [13,48], which are 2 distinguishing characteristics of problem Internet gamblers. Innovative targeted advertising strategies, including through social media, may be needed to better promote help services to target young male online gamblers experiencing problems with non-EGM forms of gambling [30,46]. A further explanation is that problem Internet gamblers may value their privacy more than problem land-based gamblers, as reflected through their choice of main gambling mode and an apparent reticence to disclose their problem gambling to those who can provide help. The privacy and lack of scrutiny afforded by the online gambling environment [49-54] may also facilitate problem denial. The relative isolation of Internet gambling means that Internet gamblers typically receive no cues from venue staff or other patrons that their gambling may be problematic [29].

However, problem Internet gamblers in this study were found to have lower levels of both gambling problems and psychological distress, and thus fewer reasons to seek help. Therefore, they may have less severe negative consequences from their gambling and may also be less likely to gamble for escape and dissociation, which are well-known motivations for EGM gambling among problem land-based gamblers [13,17]. In comparison, sports and race wagering are engaged in more often for recreational and entertainment reasons, as a hobby and a challenge. For example, a study of regular horse and EGM gamblers found that horse gamblers were motivated by positively reinforced outcomes, such as excitement and reward; in contrast, EGM players were generally responding to negatively reinforced outcomes, such as escape from emotional distress [55]. Gambling for escape and mood regulation has been endorsed in previous research as increasing risks for gambling problems [56-58]. Additionally, the predominantly young male problem Internet gamblers may have fewer financial responsibilities and be better able to sustain gambling losses without experiencing severe adverse consequences compared to problem land-based gamblers.

This study also compared use of formal online, telephone, and face-to-face help by most problematic gambling mode. Researchers have suggested that problem Internet gamblers are more likely to use online than land-based help [32-34] and this was proposed by our second hypothesis. However, an aversion to help seeking through the same medium associated with problem gambling has also been suggested, although this proposition has not been tested [11]. The current study found that use of online formal help did not differ by most problematic gambling mode. Despite the availability of diverse types of online help, including counselor-assisted therapy through live chat and email, peer support groups, and self-help tools, resources, and apps [34,59,60], online options did not appear to be more attractive to problem Internet gamblers than to problem land-based gamblers. Therefore, our second hypothesis that problem Internet gamblers are more likely to seek online help for problem gambling compared to problem land-based gamblers was not supported. In contrast, problem land-based gamblers in the current study were more likely to use land-based or telephone-based formal help compared to problem Internet gamblers. This finding may reflect their lower comfort levels with using the Internet to seek help, as reflected through their preferred gambling mode, but also greater promotion of land-based and telephone help services than online help options in the land-based venues they frequent. It may also reflect that the availability of online help options may not be well advertised or promoted to either Internet or land-based problem gamblers.

Further research is needed to explain why help-seeking rates appear to be lower among problem Internet gamblers compared to problem land-based gamblers. It may be that they have less need to seek help or that some find self-exclusion from gambling websites adequate to maintain control over gambling. Alternatively, barriers to help seeking may be different for problem Internet gamblers than those found among problem land-based gamblers, such as stigma, shame, problem denial, a belief that one can handle the problem alone, and false hope in the ability to win back losses or regain control [19,61-65]. Research with representative samples is also needed to verify results obtained.

### Limitations

Low numbers of Internet gamblers in the population necessitated a targeted approach to recruitment. Thus, although the study sample was large, it was not necessarily representative of the general population of problem Internet gamblers or of problem land-based gamblers among whom use of the Internet may be low. This convenience sampling may explain the high rates of help seeking found in this study compared to previous estimates that approximately 10% of problem gamblers in Australia seek professional help for their gambling problem [13]. Recruitment of respondents through advertisements on gambling help sites, possibly greater participation in the survey by those with higher gambling involvement, and inclusion of specific questions on each types of help (eg, gambling telephone helpline, self-exclusion, self-help), may also explain the comparatively high help-seeking rates found in the current study. A further limitation is that the help-seeking questions did not specifically ask whether the help was sought in relation to Internet or land-based gambling; this limitation could be avoided in future research. Similarly, most problematic mode of gambling and most problematic form of gambling were self-assessed rather than being ascertained through screening. Additionally, the cross-sectional nature of the study did not allow causal inferences to be drawn.

### Conclusions

Classifying problem Internet and problem land-based gamblers based on most problematic gambling mode appears an advantageous approach to analyzing their characteristics and behaviors because it removes the confounding issue that some Internet gamblers experience most problems with a land-based mode of gambling. By using this approach, this study was able to identify distinguishing characteristics of problem Internet gamblers as being more likely to be male, younger, with lower levels of psychological distress than their land-based counterparts, and to most likely have problems with sports and race wagering. Further, this approach identified lower uptake of help by problem Internet gamblers compared to problem land-based gamblers and a similarly low use of online help among the 2 groups.

Findings suggest that targeted, more innovative, and widespread efforts may be needed to increase use of gambling help by problem Internet gamblers, including through their promotion on Internet gambling websites. Internet gambling operators could also ensure that harm minimization measures, such as deposit limits, credit limits, and self-exclusion, are available and prominently advertised. Further, promotion of gambling help could be tailored to the predominantly young male profile of problem Internet gamblers and focus on risks associated with online sports and race wagering in addition to the current focus of most public health initiatives on EGM gambling. Online self-help resources should also be further developed to cater to Internet gamblers and be promoted to both Internet and land-based gamblers. However, additional research is needed to further understand why help-seeking rates appear to be lower among problem Internet compared to problem land-based gamblers. Whether this group of problem gamblers has the same need for gambling help as their land-based counterparts is not currently known.

## Abbreviations

EGM: electronic gaming machine
PGSI: Problem Gambling Severity Index
